# How Two Small Pharmacy Schools’ Competency Standards Compare with an International Competency Framework and How Well These Schools Prepare Students for International Placements

**DOI:** 10.3390/pharmacy5010014

**Published:** 2017-03-06

**Authors:** John Hawboldt, Rose Nash, Beverly FitzPatrick

**Affiliations:** 1 School of Pharmacy, Memorial University, St. John’s, NL A1B3V6, Canada; hawboldt@mun.ca; 2 Public Health and Global Health, School of Medicine, University of Tasmania, Hobart 7000, Australia; rose.mcshane@utas.edu.au

**Keywords:** international placement, pharmacy undergraduates, curriculum, standards

## Abstract

International standards of pharmacy curricula are necessary to ensure student readiness for international placements. This paper explores whether curricula from two pharmacy programs, in Australia and Canada, are congruent with international standards and if students feel prepared for international placements. Nationally prescribed educational standards for the two schools were compared to each other and then against the International Pharmaceutical Federation (FIP) Global Competency Framework. Written student reflections complemented this analysis. Mapping results suggested substantial agreement between the FIP framework and Australia and Canada, with two gaps being identified. Moreover, the students felt their programs prepared them for their international placements. Despite differences in countries, pharmacy programs, and health-systems all students acclimatized to their new practice sites. Implications are that if pharmacy programs align well with FIP, pharmacists should be able to integrate and practise in other jurisdictions that also align with the FIP. This has implications for the mobility of pharmacy practitioners to countries not of their origin of training.

## 1. Introduction

Pharmacy education, in the era of globalization, should consider international practice experience placements (PEP) for pharmacy students. International PEP can increase students’ cultural competence, enhance awareness of other health-systems, and provide exposure to diseases/medicines that may be uncommon in their respective countries. Alsharif indicated that international experiences can increase students’ respect for local, national, international, and ethnic identities [1]. Moreover, Cisneros et al., suggested that these placements can also increase students’ contributions to global healthcare [2]. Through PEP, Owen argued that students are provided with situations that enhance the knowledge and teaching gained through classic university education as they are immersed in real patient-care settings under the supervision of professional practitioners [3]. International placements should be useful as they can assist in the preparation of future pharmacists for the challenges of a multicultural and increasingly globalized world.

Students enrolled in Pharmacy courses at The University of Tasmania, Australia (UTas) and Memorial University, Newfoundland and Labrador, Canada (MUN) request varied placement experiences and sites that offer diverse community and institutional health experiences. This includes interdisciplinary involvement, patient-centred care, quality sites, experienced preceptors, and travel opportunities. Currently, UTas and MUN have a reciprocal arrangement that offers opportunities for students to complete PEP in Australia and Canada. 

The schools are located in different parts of the world, one in the southeast hemisphere, and the other in the northwest hemisphere. Both are on islands, rather than the mainland of each country. Thus, there are social, demographic and geographical similarities, as well as different challenges. UTas was established in 1890, and the Pharmacy School in 1978. MUN opened in 1925, and the School of Pharmacy in 1985. By international standards, the schools are relatively young and face challenges associated with market demands and shifting workplace expectations.

Both populations have an identifiable founder population with little immigration as compared to other states or provinces. In Tasmania, approximately 90% of its people are born in Australia and are primarily of British descent [4]. Tasmania has two major centres of population (Hobart and Launceston) on opposite side of the Island. Newfoundland started with about 20,000 settlers in 1760 with approximately 98% of the current population being of English or Irish descent [5] Newfoundland also has two major population centres (The Avalon Peninsula and the city of Corner Brook) again, also on opposites sides of the Island. Anecdotally, the three authors of this article would agree that there is also a form of discrimination against both Island populations from their mainland counterparts in the forms of jokes about their respective genetic profiles or with regards to perceived levels of intelligence. All of these factors, including a historical “Island induced” social isolation from their mainland countrymen, has led to many similarities between both Tasmania and Newfoundland.

In terms of health care, in Australia the government provides subsidised healthcare and medicines through Medicare and the Pharmaceutical Benefits Scheme in Hospitals and the Community setting. Medicare is partially funded by income tax surcharges. While Australians enjoy free public hospital services, all patients make “means tested contributions” to their other healthcare and medication costs. Individuals can get private health cover to increase their choices, get access to services, and receive subsidised care on “private services”. In Canada, there is a single payer (the provincial Government) when it comes to medicines delivered in the institutional setting. However, in the community setting, medicines may be provided by a private insurer (with or without a co-pay), exclusively by the provincial government, partially by the provincial government (with a co-pay), or the patient pays out-of-pocket. 

With regards to educational programs, the Australians provide a “degree plus professional registration” while the Canadian pharmacy programs follow a “registration upon graduation” system. A main difference between these two programs is that the majority of practice experience is provided to the Australian students after they graduate from their program under the guidance of a preceptor and intern training provider; while in Canadian programs, the practice experiences are integrated within the program at least once per academic year.

Students in UTas are able to enter their pharmacy program straight out of high school and graduate from a four year program with a Bachelor of Pharmacy. Students at MUN require a minimum one year of university with specific credits and graduate from a four year pharmacy program with a Bachelor of Science in Pharmacy. Both UTas and MUN students have raised concerns about the quality of their local placement sites, the practices they are observing, and the alignment of practice with their university studies and personal expectations. The fact that this is occurring in both schools speaks to similarities found in both programs and also supports the argument that pharmacy education around the world has more similarities than differences.

Medicine was one of the first health-care professions to attempt to develop a global competency framework, the purpose being to ensure that competencies for all physicians, despite geographic area, will be transparent, applicable, and transferable to other jurisdictions [6]. Regardless of socioeconomic, cultural, teaching, and health-systems differences between countries, the World Federation for Medical Education believes that the basic science of medicine is universal [6]. The International Pharmaceutical Federation (FIP), advocates a similar position for Pharmacy Education [6]. Thus, FIP developed the Global Competency Framework (GCF) as a mapping tool that deals with initial education and training for those who have an interest in globalizing or harmonizing the expectations of what a pharmacy practitioner should be [6]. The GCF was developed from an extensive literature review on pharmacy competencies and practice with a comparative study that identified common behaviors that should be applicable to the pharmacy workforce worldwide [6]. The GCF was intended to be used as a mapping tool that would evolve with the profession. The document consisted of four domains (e.g., pharmaceutical public health—a population focus), various competencies (e.g., health promotion) and behaviors associated with each competency [6]. This was done with the intent of providing outcomes in the training and practice of pharmacists. This in turn would therefore be useful to regulators and educators who are interested in promoting global consistency with regards to the expectation of practice for pharmacy practitioners [6].

In order for an international PEP to be effective, the educational experiences of the PEP must align with the respective programs’ expectations and learning objectives. Moreover, FIP has acknowledged, “Practitioner Development frameworks, containing a structured assembly of behavioral competencies have become increasingly popular in professional education, driven by the need for transparency in the training, development and professional recognition of healthcare professionals” [6] (p. 3). Our research questions are twofold: how comparable are the respective Australian and Canadian competencies with the FIP Framework, and how well prepared do the students from the two schools feel to complete their international placements.

## 2. Materials and Methods

To establish how well students from UTas and MUN are prepared for global pharmacy practice, we used two methods of data collection. To answer the first research question, we analysed the curriculum standards associated with each program. This was achieved by mapping the educational outcomes from both pharmacy schools against the GCF of FIP [6]. For the mapping in this study, the FIP GCF was used as a common denominator as it sets a global pharmacy standard to which the educational outcomes could be compared. Each outcome from the Australian and Canadian documents, and then the FIP GCF, was analysed for content and meaning to determine if there were matching outcomes in the documents. The individual authors from each respective country completed mapping of country specific educational outcomes with FIP. Then, the authors from both countries worked together to find the commonalities and differences between their countries, and with FIP. This involved several meetings through Skype to achieve common understandings of the outcomes and to reach consensus on the mapping. As well, numerous emails transpired between the authors on an ongoing basis throughout the analysis.

The educational outcome documents from Australia and Canada included: the Australian Pharmacy (AP) Threshold Learning Outcomes (PhLOs), the National Competency Standards (NCSs) Framework for Pharmacists in Australia (PA), The Association of Faculties of Pharmacy of Canada (AFPC) Educational Outcomes (EOs) for First Professional Degree Programs in Pharmacy in Canada, and the National Association of Pharmacy Regulatory Authorities (NAPRA) Professional Competencies (PCs) for Canadian Pharmacists at Entry to Practice [3,7,8,9]. Although each school has course objectives which are mapped against each school’s national competencies, this level of analysis was not included in this paper. Because this mapping had already been conducted, the authors were confident that the national outcome documents reflected the goals and outcomes for their individual schools. Thus, mapping was done at the national level for the purpose of this paper. 

To address the second research question we went directly to students who were participating in an international PEP. This allowed a more complete understanding of the meaning of the outcomes and how they related to international standards, as it gave us a student perspective of what they were learning. Four students from UTas and two students from MUN participated in an international PEP, where they spent six weeks in each other’s Schools. The students had completed three years of their programs before the placements. We endeavoured to learn what the students experienced in their programs and how they used this knowledge in their international PEP. 

As part of the PEP the students wrote reflections, based on specific guidelines developed by one of the authors who has experience in education and qualitative research, about the commonalities and differences they observed and experienced during their placements, how this related to the education they received in their pharmacy programs, and whether they thought they were prepared to practise globally. See Table 1 for reflection guidelines. These data were first cycle structurally coded for concepts to examine commonalities, differences, and relationships, and then second cycle pattern coded to search for major themes and explanations in the data [10]. First cycle coding is the initial coding where broad categories are developed and second cycle coding follows to more fine tune the categories and develop specific concepts. We used structural coding for the initial analysis which examined how the students answered the questions and to establish a framework for the results. In our second cycle of coding we looked for patterns to develop themes and explanations. Ethics as per the Helsinki declaration was obtained for all participants.

## 3. Results

### 3.1. Mapping Results

The top-level domains of each document are listed in Table 2. Where possible, mapping proceeded to the furthest sub-domain in each document. 

After the mapping was completed, only two gaps were evident. First, AFPC did not map against FIP or any of the Australian documents regarding compounding of medicines. The specific FIP competency missed included:

“Prepare pharmaceutical medicines (e.g., extemporaneous, cytotoxic medicines), determine the requirements for preparation (calculations, appropriate formulation, procedures, raw materials, equipment etc.).

Compound under the good manufacturing practice for pharmaceutical (GMP) medicines.”

The second gap was in the FIP domain of organization and management competencies. This was again exclusively with one competency document, the AP PhLOs. The gap determined was in:

“Acknowledge the organizational structure.

Effectively set and apply budgets.

Ensure appropriate claim for reimbursement.

Ensure financial transparency.

Ensure proper reference sources for service reimbursement.”

Other than these two gaps, the mapping revealed consistency across all domains in all of the documents. Moreover, these particular gaps were not evident with the NCS and NAPRA documents. Most importantly, improving patient care was a consistent theme present in all competencies.

### 3.2. Student Reflections

The students thought that similar topics were taught in both programs, but the emphases placed on specific concepts within these topics differed in the two schools. For example, while both groups of students studied therapeutics, the students from UTas thought the MUN students spent more time learning about medications and their effects on patients than the patient being the centre of care. One student stated that MUN had a drug-oriented approach with “goals of care for each single medication”, whereas UTas had a patient oriented approach with “goals of care for the patient as a whole”. The UTas students thought MUN was more academically focused on facts and theory, while the Utas program concentrated more on practical outcomes such as patient interaction, critical thinking, and communication skills. Instructors from both Schools discussed the student comments and considered them as teaching and learning points for future instruction. 

Students from both schools participate in experiential learning during their programs, and all students thought there were similar expectations from the preceptors in both countries. MUN students participate in experiential placements each year of their program, some of which are six weeks in duration. UTas students have fewer and shorter placements, but participate in fortnightly hospital visits where they are encouraged to interact with actual patients. Students from both countries discussed advantages and disadvantages of the different types of experiential learning. For example, one student from UTas thought that “at the end of three weeks a student may just be feeling comfortable, then they are moved to the next placement”, but at MUN with the six-week placement, students get the opportunity to “become better accustomed to the workplace”. In both countries, students reported that medication reconciliation is important and made the observation that they observed mutual respect between pharmacists and physicians.

All the students thought they were adequately prepared to complete their PEP. They had to get used to different drugs and brand names, and some differences in what constituted controlled substances. Students also had to learn about the different healthcare systems in each country. However, the differences between the two countries were not surmountable. One student said it this way, “the programs are similar in what we learn, especially therapeutically, this enabled me to apply that knowledge here and effectively work as I would have at home, with the same expectations”.

## 4. Discussion

There was strong agreement regarding standards and competencies among the documents. The Australian (PhLOs and NCS) and Canadian (AFPC and NAPRA) standards aligned strongly with the FIP standards, and aligned well with each other. The PhLOs and AFPC EOs did not match the FIP GCF with respect to two particular “practical” competencies, those being compounding and management principles. This is despite the fact that these principles are provided in both curricula and are everyday activities of practising pharmacies. These competencies were covered by the respective documents (NAPRA PCs and NCS for AP), which primarily deal with the practice component of a pharmacist’s training.

The strong agreement among the respective documents is also reflected in the students’ written reflections. With the exception of a few key points, such as patient-focus versus patient–drug focus, and some regulatory issues, all the students appeared to integrate well into the individual practice experience. This was evident from the positive student and preceptor comments. Further, no students or their respective preceptors identified adjustment issues, even though both countries have slightly different health-systems and pharmacy programs. Despite these health-system differences, the students accommodated to their new practice sites with minimal discomfort. In addition, the students’ ability to practise effectively in another jurisdiction was not affected by the differences between the two programs in how and when students participate in their experiential learning.

There are reasons beyond the mapping and student reflections that could contribute to the ease with which these students completed their PEP. Possibilities include language (both are primarily English speaking locations), ethno-cultural similarities (both have highly homogenized, traditionally Anglo-Saxon populations), and although slightly different, the health-systems are still first world, and would have a high degree of similarity in the services they provide.

Limitations of this paper include the small sample size for the student reflections, but the student comments should be used judiciously for generalization. They provide an example of student experiences in international placements that are not meant to be universal, but complement the document analysis to provide context and meaning to our comparison.

Since this positive experience, the MUN School of Pharmacy has increased its intake of international students to include students from Australia and the United States. Moreover, MUN students continue to participate in PEP in Australia and are now in other countries such as the United States. UTas continues to send student to MUN and elsewhere. Both schools see the value in the internationalization of their practice experiences in that not only do the students see how another healthcare system provides for its citizens, they also are exposed to the unique role that the pharmacists play in that system. It is these learnings that they can take back to their respective jurisdictions and act as agents of change.

## 5. Conclusions 

Despite differences in countries, pharmacy programs, and health-systems the students from both countries successfully completed their international practice experience placements at their new practice sites. This is encouraging as it implies that if the standards and competencies of pharmacy programs have good alignment with the international standards of GCF, then PEP will not be affected by the differences in students’ education at individual schools. Moreover, this has implications for the mobility of pharmacy practitioners to countries not of their origin of training. More research needs to be conducted to determine if these findings would apply to practising pharmacists who might like to transition to other countries. 

## Figures and Tables

**Table 1:** Guidelines for Student Reflections.

Write about the SPE in the second country and relate it to the pharmacy program you received in your own country. Include how well the pharmacy program in your country prepared you for the SPE in the second country. Suggested guidelines are below. Please add anything else that comes to mind, including examples of specific incidences.
Write about: Similarities Differences What was easy in the second country What was difficult in the second country What it was (specifically) in your program that made it possible/practicable for you to do a SPE in a second country The most important learning(s) you got from your program The most important learning(s) in the second country What you learned during the SPE that was new and that you might not be able to put into practice in your own country

**Table 2:** Top Level Domains of the Australian Pharmacy (AP) Threshold Learning Outcomes (PhLOs) and CSs, the Canadian Association of Faculties of Pharmacy of Canada (AFPC) EDs and National Association of Pharmacy Regulatory Authorities, Professional Competencies (NAPRA PCs), and the International Pharmaceutical Federation, Global Competency Framework (FIP GCF) [3,6,7,8,9].

Jurisdictions	Top-Level Domains
Australian Pharmacy Threshold Learning Outcomes	1. “Demonstrate professional behaviour and accountability in the commitment to care for and about people”. 2. “Retrieve, critically evaluate, and apply evidence in professional practice”. 3. “Demonstrate team and leadership skills to deliver safe and effective practice”. 4. “Make, act on, and take responsibility for clinically, ethically, and scientifically sound decisions”. 5. “Communicate in lay and professional language, choosing strategies appropriate for the context and diverse audiences”. 6. “Reflect on current skills, knowledge, attitudes, and practice; planning and implementing for ongoing personal and professional development”. 7. “Apply pharmaceutical, medication, and health knowledge and skills: -Within their scope of practice, in the assessment of individual health status and medication needs, and where necessary, develop, implement and monitor management plans in consultation with patients/clients and other health professionals to improve patient outcomes, and -To promote and optimise the health and welfare of communities and/or populations”. 8. “Formulate, prepare, and also supply medications and therapeutic products”.
National Competency Standards Framework for Pharmacists in Australia	Domain 1: “Professional and ethical practice addressing the legal, ethical and professional responsibilities of pharmacists”. Domain 2: “Communication, collaboration, and self-management required to communicate effectively with consumers and colleagues, and build and maintain cooperative working relationships within the healthcare team”. Domain 3: “Leadership and management relating to how pharmacists apply management and organisational skills ensuring the effective and efficient delivery of pharmacy services”. Domain 4: “Review and supply prescribed medicines for accurate and timely supply of prescription medicines, including extemporaneously prepared products”. Domain 5: “Prepare pharmaceutical products required for the extemporaneous preparation of single or multiple units of a medicine for immediate issue and/or use by a specific consumer”. Domain 6: “Deliver primary and preventative health care addressing the role pharmacists have in encouraging and assisting individual and groups of consumers to take responsibility for their own health”. Domain 7: “Promote and contribute to optimal use of medicines addressing aspects of clinical practice directed that ensures the safe and appropriate management of medicines”. Domain 8: “Critical analysis, research, and education addressing the capability of pharmacists to analyse and synthesise information from medical and pharmaceutical literature”.
Association of Faculties of Pharmacy of Canada Educational Outcomes for First Professional Degree Programs in Pharmacy in Canada,	Care Provider: “Pharmacy graduates use their knowledge, skills, and professional judgement to provide pharmaceutical care and manage a patient’s medication and overall health needs”. Communicator: “Pharmacy graduates communicate with diverse audiences, using various strategies that consider the situation, intended outcomes of the communication, and the target audience”. Collaborator: “Pharmacy graduates work collaboratively with teams to provide effective health care and to fulfil their professional obligations to the community at large”. Manager: “Pharmacy graduates use management skills to optimize patient care ensuring the safe and effective distribution of medications, and efficient use of health resources”. Advocate: “Pharmacy graduates advance the health and well-being of individual patients, communities, and populations, and support pharmacists’ professional roles”. Scholar: “Pharmacy graduates apply the knowledge and skills required to be a medication therapy expert, and are able to master, generate, interpret, and disseminate pharmaceutical and pharmacy practice knowledge”. Professional: “Pharmacy graduates honour their roles as self-regulated professionals through both individual patient care and fulfilment of their professional obligations to the profession, and the community”.
National Association of Pharmacy Regulatory Authorities Professional Competencies (PCs) for Canadian Pharmacists at Entry to Practice	Ethical, Legal and Professional Responsibilities: “Pharmacists practise within legal requirements, demonstrate professionalism, and uphold professional standards of practice, codes of ethics, and policies”. Patient Care: “Pharmacists, in partnership with the patient and in collaboration with other health professionals, meet the patient’s health and drug-related needs to achieve the patient’s health goals”. Product Distribution: “Pharmacists ensure accurate product distribution that is safe and appropriate for the patient”. Practice Setting: “Pharmacists oversee the practice setting with the goal of ensuring safe, effective and efficient patient care”. Health Promotion: “Pharmacists use their expertise to advance the health and wellness of patients, communities and populations”. Knowledge and Research Application: “Pharmacists access, retrieve, critically analyse and apply relevant information to make evidence-informed decisions ensuring safe and effective patient care”. Communication and Education: “Pharmacists communicate effectively with patients, the pharmacy team, other health professionals, and the public, providing education when required”. Intra and Inter-Professional Collaboration: “Pharmacists work in collaboration with the pharmacy team and other health professionals to deliver comprehensive services, make best use of resources, and ensure continuity of care in order to achieve the patient’s health goals”. Quality and Safety: “Pharmacists collaborate in developing, implementing, and evaluating policies, procedures, and activities that promote quality and safety”.
International Pharmaceutical Federation Global Competency Framework	1. “Pharmaceutical Public Health Competencies where the pharmacist will be involved in such activities as health promotion and medicines information/advice”. 2. “Pharmaceutical Care Competencies whereby pharmacists will assess effective use of medicines, compound medicines, dispense medicines, monitor medicine therapy, and provide patient consultation/diagnosis”. 3. “Organization and Management Competencies where concepts such as budget/reimbursement, human resources management, improvement of service, procurement, supply chain, supply management and work place management are provided”. 4. “Professional/Personal Competencies where pharmacists will apply concepts such as communication skill improvement, the importance of continuing professional development, legal/regulatory practice, professional/ethical practice, quality assurance/research in the workplace and self management”.
